# Supplementary figures and images for: An in vitro ovarian explant culture system to examine sex change in a hermaphroditic fish
Source: PeerJ. 2020 Nov 11;8:e10323. doi: 10.7717/peerj.10323 (PMC7666549; doi:10.7717/peerj.10323)

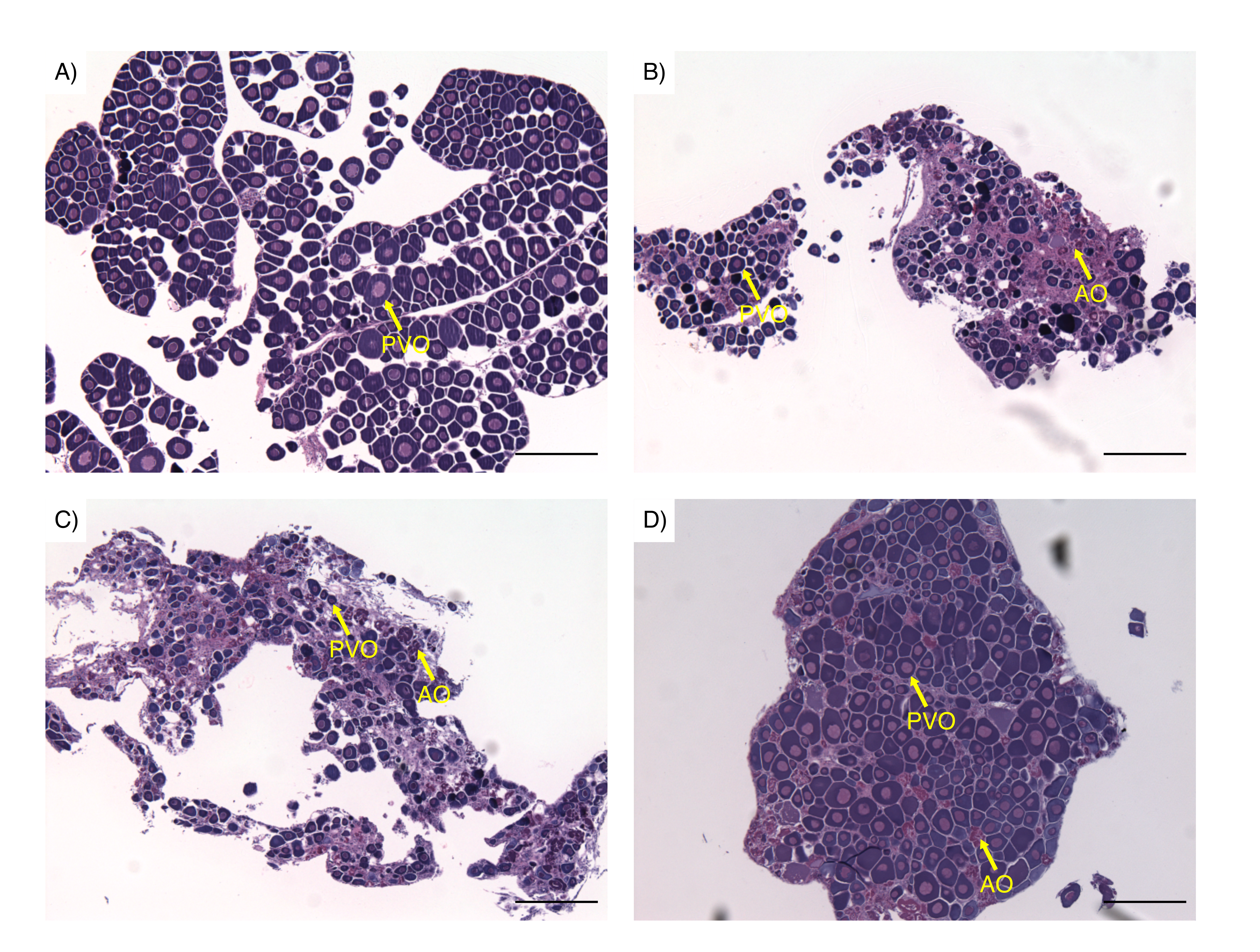

Supplement: Supplemental Information 1 — (A) Ovarian tissue preserved for histological analysis as day 0 reference tissue. (B) Ovarian tissue treated with 100 ng/mL of 17 β-estradiol. (C) Ovarian tissue treated with 10 ng/mL of 11-ketotestosterone. (D) Ovarian tissue treated with 100 ng/mL of cortisol. Abbreviations: previtellogenic oocytes (PVO), atretic oocytes (AO). [file peerj-08-10323-s001.png]
